# An engineered niche delineates metastatic potential of breast cancer

**DOI:** 10.1002/btm2.10606

**Published:** 2023-09-29

**Authors:** Sophia M. Orbach, Christian Y. DeVaull, Elizabeth J. Bealer, Brian C. Ross, Jacqueline S. Jeruss, Lonnie D. Shea

**Affiliations:** ^1^ Department of Biomedical Engineering University of Michigan Ann Arbor Michigan USA; ^2^ Department of Pathology University of Michigan Ann Arbor Michigan USA; ^3^ Department of Surgery University of Michigan Ann Arbor Michigan USA; ^4^ Department of Chemical Engineering University of Michigan Ann Arbor Michigan USA

**Keywords:** breast cancer, early detection, engineered diagnostics, gene signature

## Abstract

Metastatic breast cancer is often not diagnosed until secondary tumors have become macroscopically visible and millions of tumor cells have invaded distant tissues. Yet, metastasis is initiated by a cascade of events leading to formation of the pre‐metastatic niche, which can precede tumor formation by a matter of years. We aimed to distinguish the potential for metastatic disease from nonmetastatic disease at early times in triple‐negative breast cancer using sister cell lines 4T1 (metastatic), 4T07 (invasive, nonmetastatic), and 67NR (nonmetastatic). We used a porous, polycaprolactone scaffold, that serves as an engineered metastatic niche, to identify metastatic disease through the characteristics of the microenvironment. Analysis of the immune cell composition at the scaffold was able to distinguish noninvasive 67NR tumor‐bearing mice from 4T07 and 4T1 tumor‐bearing mice but could not delineate metastatic potential between the two invasive cell lines. Gene expression in the scaffolds correlated with the up‐regulation of cancer hallmarks (e.g., angiogenesis, hypoxia) in the 4T1 mice relative to 4T07 mice. We developed a 9‐gene signature (*Dhx9*, *Dusp12*, *Fth1*, *Ifitm1*, *Ndufs1*, *Pja2*, *Slc1a3*, *Soga1*, *Spon2*) that successfully distinguished 4T1 disease from 67NR or 4T07 disease throughout metastatic progression. Furthermore, this signature proved highly effective at distinguishing diseased lungs in publicly available datasets of mouse models of metastatic breast cancer and in human models of lung cancer. The early and accurate detection of metastatic disease that could lead to early treatment has the potential to improve patient outcomes and quality of life.

**Translational Impact Statement**

Metastatic breast cancer is often not diagnosed until secondary tumors have become macroscopically visible and millions of tumor cells have invaded distant tissues. We used a porous, polycaprolactone scaffold, that serves as an engineered metastatic niche, to distinguish early metastatic disease from invasive, non‐metastatic cancer. The early and accurate detection of metastatic disease that could lead to early treatment has the potential to improve patient outcomes and quality of life.

## 1 INTRODUCTION

Approximately 4% of women in the United States will develop metastatic breast cancer, which has a 10‐year survival rate of less than 15%.^1^, ^2^, ^3^ A major inhibitory factor toward improving these outcomes is an inability to detect metastatic disease prior to macroscopic secondary tumor formation, at which point millions of tumor cells have colonized distant sites.^4^, ^5^, ^6^ Secondary tumor formation requires cancer cells to complete a complex series of events referred to as the metastatic cascade.^7^ Cancer cells must invade neighboring tissue from the primary tumor, intravasate, survive in circulation, extravasate to a secondary site, and then colonize and proliferate at that site to form a metastatic tumor. Identification of systemic markers correlating with these events could be the key to early detection, thus earlier treatment, of metastatic breast cancer. Furthermore, an ability to distinguish metastatic from nonmetastatic disease could improve patient‐specific therapy to avoid over‐ or under‐treatment.

Currently, in clinical disease, the primary tumor is analyzed following surgical resection and is used to assess the risk of recurrence. However, the analysis of these tumors has severe limitations, as the primary tumor microenvironment can be distinct from the metastatic environment.^8^, ^9^ Liquid biopsy has been increasingly employed for detection of metastatic cancer.^6^, ^7^, ^10^, ^11^ Elevation of blood biomarkers, such as circulating tumor cells, cell‐free circulating tumor DNA, and exosomes, have been correlated with increased risk of metastatic disease.^6^ Yet, reports show high variability and inconsistency in the clinical interpretation of these metrics. Furthermore, as only ~0.01% of circulating tumor cells have metastatic potential,^6^ liquid biopsy has limited capacity to identify invasive cancers with immediate risk for metastatic tumor formation. More recent research studies have probed blood for immune cell (e.g., neutrophil‐to‐lymphocyte ratio) and gene‐based signatures to classify disease stage and outcomes.^6^ While such signatures show promise in delineating cancer from benign lesions or early‐stage from late‐stage disease,^12^, ^13^, ^14^, ^15^, ^16^ blood analyses often misrepresent tissue cell populations and phenotypes.^6^ As metastatic development is dependent on the local microenvironment, these analyses do not identify biomarkers associated with the metastatic niche.

A microenvironment‐based signature has the potential to identify the initiation of metastases prior to the arrival of cancer cells at a secondary site. Tumor signaling induces localized changes that prime the microenvironment to support metastatic development.^17^ Once this pre‐metastatic niche has developed, secondary tumors can develop from aggressive circulatory cells or dormant cells that survived treatment.^18^ Without the pre‐metastatic niche, the likelihood of metastasis drastically decreases, even with high levels of circulating tumor cells.^18^ Currently, the assessment of endogenous tissues for biomarkers of metastatic disease occurs only after tumors have been detected due, in part, to the risk of biopsy for vital organs. Engineered diagnostics are being developed to identify peripheral biomarkers that would be present within visceral organs typical of metastasis (e.g., lungs and livers).^6^ Such engineered diagnostics in breast cancer have typically been functionalized with signaling molecules to a specific microenvironment (primarily bone)^19^, ^20^, ^21^, ^22^ or loaded with drugs as a therapeutic alternative.^23^, ^24^ We have designed a naked, porous, polycaprolactone scaffold that acts as a synthetic metastatic niche in mouse models of breast and pancreatic cancer.^25^, ^26^, ^27^, ^28^, ^29^, ^30^ The scaffolds, when implanted subcutaneously, recruit aggressive populations of tumor cells prior to their detection at native metastatic sites.^27^, ^28^ This recruitment was marked by concomitant changes in immune cell types such as an increase in neutrophils in breast cancer and increases in monocytes and CD4+ T cells in pancreatic cancer.^28^, ^29^, ^31^ Analysis of the microenvironment at the scaffold more successfully predicted disease progression and recurrence than either the blood or primary tumor.^28^, ^29^

We employed the scaffold technology to analyze the microenvironment and determine its capacity to delineate metastatic cancer from nonmetastatic disease. Murine cell lines 67NR, 4T07, and 4T1 were derived from a single spontaneous breast tumor in a BALB/cfC3H mouse, each correlating with a different stage of the metastatic cascade.^32^, ^33^, ^34^ The 67NR cell line is nonmetastatic, tumor cells do not invade beyond the primary location. The invasive 4T07 cell line is also considered nonmetastatic; tumor cells are identified in distant tissues but do not colonize nor proliferate at these sites. The 4T1 cell line is highly metastatic, primarily to the lung. These cell lines have been widely studied to understand the biology of cancer cells throughout the metastatic cascade.^35^, ^36^, ^37^, ^38^, ^39^, ^40^, ^41^ Herein, we investigated the impact of these sister cell lines on the microenvironment of the native and synthetic metastatic niche to distinguish metastatic from nonmetastatic disease. We developed a 9‐gene signature that effectively identifies metastatic potential of triple‐negative breast cancer throughout the progression of disease.

## 2 RESULTS

We aimed to identify a microenvironment‐based multivariate signature that delineates metastatic potential in early breast cancer. As our research group has previously published a scaffold‐derived 10‐gene signature for metastatic progression and therapeutic resistance,^29^ we began by applying a known signature to this system. Interestingly, the expression of each gene in the 10‐gene signature (*Bmp15* was not identified) was highly correlated between scaffolds from the 4T07 and 4T1 mice (Figure S1) indicating that the previous signature was limited in its ability to delineate metastatic potential of invasive cancer. Therefore, we initiated new analyses centered on differentiating metastatic and nonmetastatic breast cancer.

### 2.1 The primary tumor microenvironment poorly correlates with metastatic potential

**FIGURE 1:**
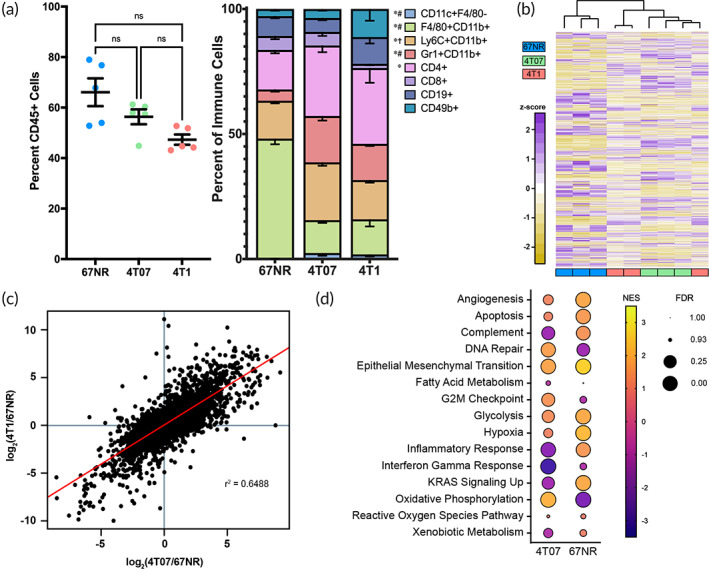
Cellular and genetic distributions of 67NR, 4T07, and 4T1 primary tumors. (a) Immune cell populations in the primary tumor are similar between the 4T07 and 4T1 tumors. Measured populations were total immune cells (CD45+), dendritic cells (CD11c + F4/80‐), macrophages (F4/80 + CD11b+), monocytes (Ly6C + CD11b+), neutrophils (Gr1 + CD11b+), CD4+ T cells (CD4+), CD8+ T cells (CD8+), B cells (CD19+), and NK cells (CD49b+), *p < 0.05 between 67NR and 4T07 tumors, ^#^ *p* < 0.05 between 67NR and 4T1 tumors, ^†^ *p* < 0.05 between 4T07 and 4T1 tumors. (b) Gene expression of the primary tumors separates 67NR (blue) from 4T07 (green) and 4T1 (red) tumors. (c) Correlation between changes in expression between both invasive tumors and the 67NR tumors. (d) Hallmark signaling pathways between 4T1 tumors and either 67NR or 4T07 tumors as determined through Gene Set Enrichment Analysis, NES = normalized enrichment score, FDR = false discovery rate, *n* = 3 (panels b‐d) or 5 (panel a) mice per condition.

Primary tumors are often the most accessible disease‐relevant tissue, and analysis of this tissue is used for most clinical decision‐making. Hence, we first analyzed the immune cell composition and gene expression within the 67NR, 4T07, and 4T1 primary tumors. Fourteen days post‐inoculation, the 67NR tumors had a distinct composition of innate immune cells (Figure 1a). Specifically, F4/80 + CD11b + macrophages comprised 48% of the immune cells in the 67NR tumors, which was approximately 3.5‐fold higher than the invasive counterparts. Furthermore, the 67NR tumors had essentially no CD11c + F4/80‐ dendritic cells (0.16%), approximately four‐fold fewer Gr1 + CD11b + neutrophils, and approximately two‐fold fewer CD4+ T Cells. High densities of tumor‐associated macrophages, neutrophils, and CD4+ T cells have typically been associated with poor prognosis in breast cancer.^42^, ^43^, ^44^ These data indicate that neutrophil and lymphocyte invasion may play a more predominant role in metastatic progression. In contrast, immune compositions of 4T07 and 4T1 tumors were highly comparable, with similar fractions of CD11c + F4/80‐ dendritic cells, F4/80 + CD11b + macrophages, and CD4+ T cells. Although there were 3.2‐fold more CD8+ T cells in the 4T07 tumors (*p* = 0.16) and 3.1‐fold more CD49b + natural killer (NK) cells in the 4T1 tumors (*p* = 0.34), these differences were not significant due to increased variability of these low‐density populations.

The distinct differences between the 67NR tumor and 4T07/4T1 tumors in the immune cells were also seen in gene expression. Specifically, 67NR samples had 4364 and 3298 differentially expressed genes (adjusted *p*‐value <0.05 and fold change >2.0) relative to 4T07 and 4T1 samples, respectively (Figures 1b and S2). These differences were extensive and the 67NR primary tumors were isolated by the dendrogram. There were 1469 such genes between the 4T07 and 4T1 samples and overall gene expression did not separate 4T07 and 4T1 primary tumors by dendrogram. Unsurprisingly, a strong correlation was observed between gene expression of the invasive tumors relative to 67NR tumors (Figure 1c), which was further enhanced when only differentially expressed genes were considered (*r* ^2^ = 0.857; Figure S3). Merely six genes were up‐regulated in 4T07 tumors and down‐regulated in 4T1 tumors (*Aspg*, *Cmpk2*, *Csn1s1*, *Kcnh2*, *Phex*, *Speg*); four genes were down‐regulated in 4T07 tumors and up‐regulated in 4T1 tumors (*Acsbg1*, *Fam167a*, *Nhs*, *Ppp1r26*). Interestingly, the most differentially expressed genes between each tumor directly correlated with the most differentially expressed genes in the cultured cell lines, suggesting these changes were a result of differences in the cell lines rather than the microenvironment (Figure S4).

We further analyzed enriched pathways in primary tumors to assess if gene expression correlated with biological functions that indicate metastatic potential. Eighty‐seven pathways were significantly up‐regulated (FDR *q*‐value < 0.10) in 4T1 relative to both 67NR and 4T07 tumors which largely correlated to cell development (22 pathways) and cell structure and binding (43 pathways; Tables S1–S3). Among the hallmark gene sets (Figure 1d), most pathways exhibited opposing trends between 4T07 and 67NR tumors relative to 4T1 (e.g., complement, KRAS signaling) or were comparable between 4T1 and the nonmetastatic tumors (e.g., reactive oxygen species pathway, apoptosis). A noticeable exception is the down‐regulation of the interferon‐gamma response in the 4T1 tumors, which is consistent with our previous reports identifying anti‐cancer activity associated with interferon signaling.^45^, ^46^ Overall, these results indicate clear differences in the primary tumors; however, these differences are indicative of the inoculated cells rather than the microenvironment.

### 2.2 Systemic immune cell distributions identify invasive cancers

Immune cells are analyzed clinically throughout therapy, often in the blood, and are used as diagnostic indicators of cancer progression (e.g., the neutrophil‐to‐lymphocyte ratio increases in advanced disease).^47^ Therefore, we next investigated the immune cell distribution in the lungs (native metastatic niche), spleens (blood surrogate^48^, ^49^), and scaffolds (engineered niche) toward identifying metastatic potential.

**FIGURE 2:**
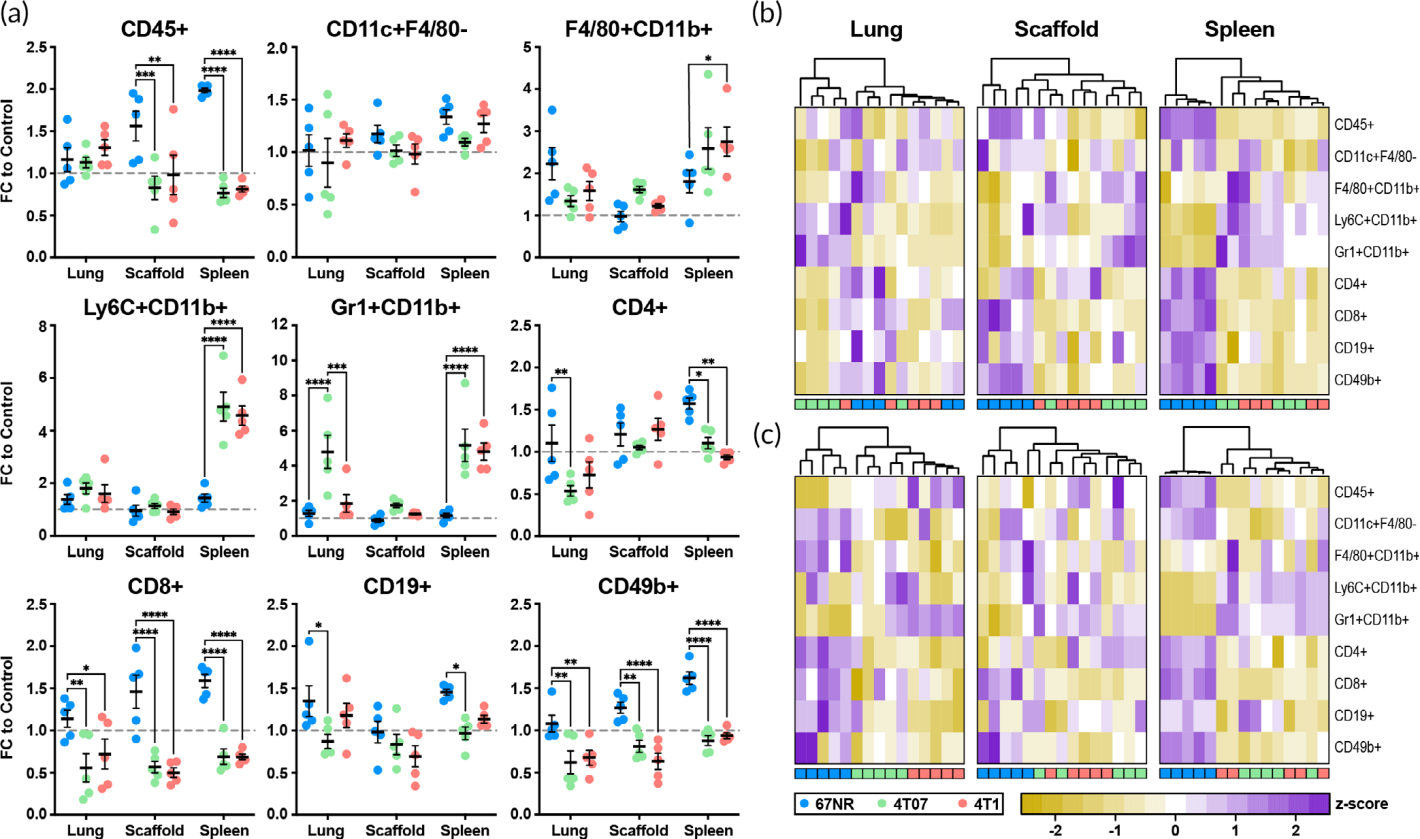
Changes to immune cell populations in response to 67NR, 4T07, and 4T1 tumor models. (a) Diseased‐induced changes to the immune response 7 days after tumor inoculation in the lung, scaffold, and spleen, blue = 67NR, green = 4T07, red = 4T1. Measured populations were total immune cells (CD45+), dendritic cells (CD11c + F4/80‐), macrophages (F4/80 + CD11b+), monocytes (Ly6C + CD11b+), neutrophils (Gr1 + CD11b+), CD4+ T cells (CD4+), CD8+ T cells (CD8+), B cells (CD19+), and NK cells (CD49b+). Clustering of these populations at (b) 7‐days and (c) 14‐days post‐inoculation did not identify metastatic potential in the scaffold or spleen, *n* = 5 mice per condition, *p < 0.05, **p < 0.01, ***p < 0.001, ****p < 0.0001.

In these cancer models, at day 7 (pre‐metastatic disease in 4T1), the populations of total immune cells (CD45+), CD8+ T Cells, and NK Cells (CD49b+) were approximately two‐fold higher (*p* < 0.05) in the scaffolds from 67NR‐treated mice than 4T07 or 4T1 mice (Figure 2a). Yet, each immune cell population was comparable between the 4T07 and 4T1 mice in the scaffolds and the spleen. Collective analysis of the immune cell populations showed that both the scaffold and the spleen could differentiate noninvasive disease (67NR), yet not metastatic cancer (4T1) from the invasive, nonmetastatic counterpart (4T07) (Figure 2b). At day 14 (early metastatic disease in 4T1), the immune cell panels in the scaffold and spleen were similarly unable to delineate 4T07 from 4T1 mice (Figure 2c). Combined, these data indicate that immune cells in the peripheral tissues (scaffolds and spleens) are altered in invasive cancer, but do not correlate with metastatic development.

Interestingly, in the lungs on day 7, each population of adaptive immune cells had significantly greater abundance in the 67NR mice (Figure 2a) relative to 4T07 or 4T1 mice. Furthermore, neutrophils were 2.6‐fold higher in lungs from 4T07 mice than 4T1 mice. Despite these differences, a dendrogram of the collective immune panel of the lungs was unable to distinguish the cell types (Figure 2b). On day 14, collective analysis of the immune cells at the lung successfully identified metastatic potential (Figures 2c and S5). These findings coincide with the arrival of tumor cells at this time point,^45^ and may reflect contributions of both systemic immune dysregulation and local changes induced by tumor cells. However, no macroscopic tumors are observed at day 14, thus, there would be no clinical indication to biopsy the lungs, suggesting the immune cell composition may have limited translational capacity in the identification of metastatic potential.

### 2.3 Gene expression changes at the scaffold select for cancer‐related signaling

**FIGURE 3:**
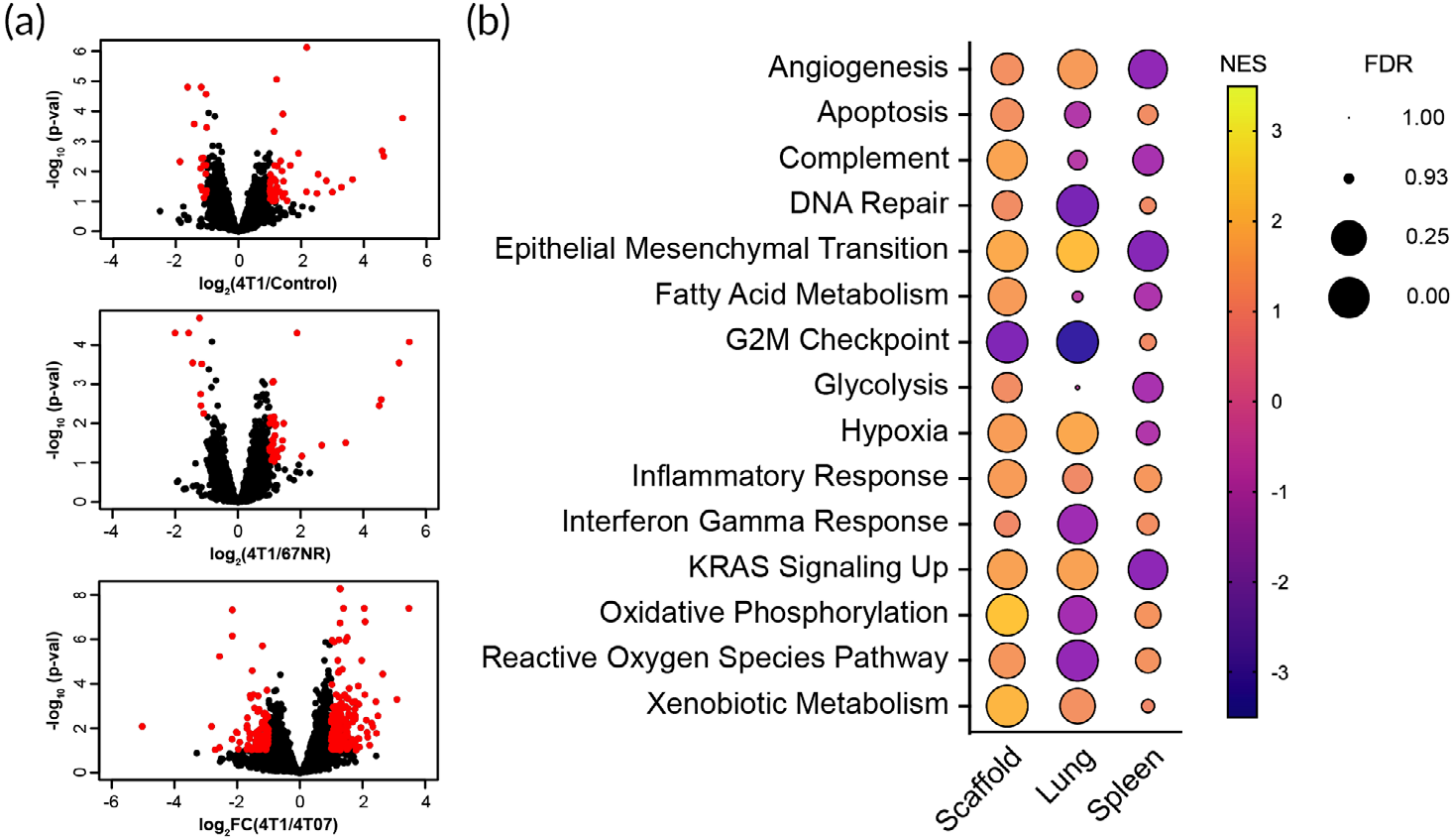
Gene expression and correlated signaling pathways in the scaffold, lung, and spleen. (a) Volcano plots of the differentially expressed genes between scaffolds derived from 4T1 mice and those derived from the healthy control, 67NR, and 4T07 mice. Red dots correlate to genes with adjusted *p*‐values <0.10 and fold change >2.0. (b) Hallmark signaling pathways in the scaffold, lung, and spleen between the 4T1 (positive) and 4T07 (negative) scaffolds as determined through gene set enrichment analysis, NES = normalized enrichment score, FDR = false discovery rate, *n* = 3 mice per condition.

We next probed gene expression of each tissue at day 14 to identify if the transcriptome of the microenvironment can differentiate invasive, nonmetastatic disease from metastatic cancer. Approximately 10‐fold fewer differentially expressed genes (fold change >2 and *p* < 0.1) were observed in the spleen between the 4T1 and 4T07 mice than between 4T1 mice and the other conditions (Figure S6). In contrast, the number of differentially expressed genes in the lung between 4T1 and 4T07 mice was approximately five‐fold higher than between the 4T1 mice and either the healthy control or 67NR mice. These findings were consistent with the immune cell distribution at day 14, where cell composition in the lungs could separate the two invasive cancers, yet the spleens could not. Interestingly, the trends in the scaffolds mirrored the responses in the lungs,^45^ although the overall differential expression between conditions was on a smaller scale (Figure 3a). In both the scaffold and lung, gene expression was uncorrelated to the inoculated cell lines indicating that differential expression corresponded with the microenvironment (Figure S7). Scaffolds from 4T07 and 4T1 mice had more differently expressed genes than the 4T1 mice and either the control (4.3‐fold) and 67NR (6.4‐fold) mice. These data suggest that, although immune cell distributions at the scaffold cannot delineate metastatic disease, gene expression of the immune and stromal cell phenotypes may identify metastatic disease.

Investigating enriched cancer pathways identified that most of the relevant hallmark gene sets were up‐regulated in the 4T1 scaffolds relative to the 4T07 scaffolds (Figure 3b and Table S4). A notable exception was the G2M checkpoint pathway, which was also strongly down‐regulated in the 4T1 lungs, and likely a result of proliferation dysregulation in cancer. Interestingly, when comparing enriched pathways in the spleen for the two invasive cell lines, angiogenesis, epithelial mesenchymal transition, and KRAS signaling were all down‐regulated in the 4T1 samples. As each of these pathways is critically important to the development of metastasis, these data further indicate that the spleen (as a surrogate for the blood) does not capture critical elements of cancer biology. Additionally, despite fewer overall differentially expressed genes in the scaffold, these changes were consistent with the increased metastasis of the 4T1 cells demonstrating the power of the scaffold transcriptome.

### 2.4 A scaffold‐derived 9‐gene signature isolates metastatic cancer

**FIGURE 4:**
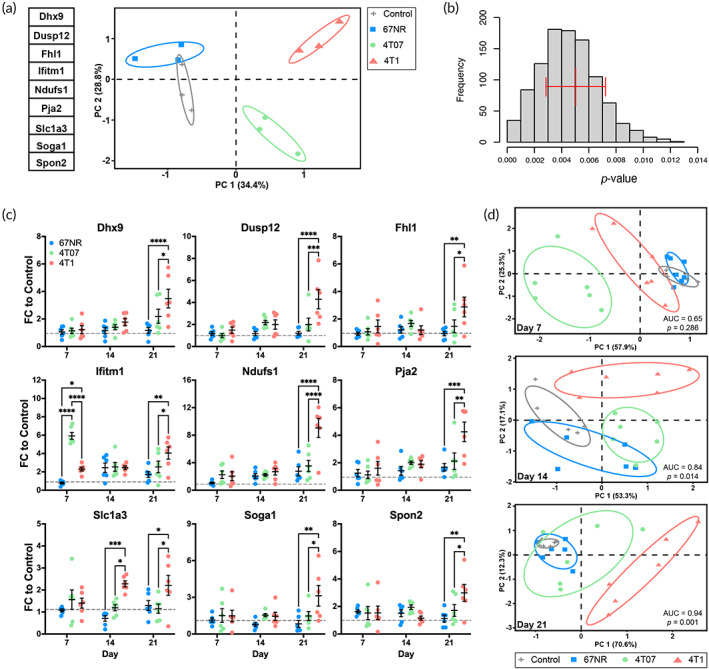
A 9‐gene signature identifies metastatic breast cancer. (a) PCA plot of the RNA‐sequencing data for the signature at day 14 in the scaffold. (b) The significance of the signature was calculated by comparing the separation of the 4T1 centroid from the other conditions to the separation using 1000 random permutations of genes *(p*‐value) repeated 1000 times (frequency). The *p*‐value indicates the fraction of gene sets that obtained separation within one standard deviation of the signature. Red bars indicate mean ± standard deviation. (c) Gene expression of each gene in the signature relative to control as determined by PCR on day 7 after tumor inoculation. Gray dotted line is control. (d) PCA plots of PCR data for the signature on the scaffold at days 7, 14, and 21. Ellipses correspond to one standard deviation, *n* = 3 (panels a, b) or 6 (panels c, d) mice per condition, *p < 0.05, **p < 0.01, ***p < 0.001, ****p < 0.0001.

We applied the mixOmics package^50^ to identify a gene set that separates scaffolds explanted from 4T1 mice relative to healthy, 67NR, and 4T07 mice (Figure 4a). The resulting microenvironment‐based signature was comprised of *Dhx9*, *Dusp12*, *Fhl1*, *Ifitm1*, *Ndufs1*, *Pja2*, *Slc1a3*, *Soga1*, and *Spon2*. PCA plots effectively isolated the 4T1 samples with 100% accuracy and an average distance from the other conditions (Control, 67NR, 4T07) of 4.05 ± 0.72 (mean ± SD).

As reports have indicated that 60% of published breast cancer signatures cannot predict outcomes better than random,^51^ we calculated a *p*‐value for our signature by comparing the average distance between groups on the PCA plots to 1000 random permutations of 9 genes. When this process was repeated 100 times, none of the 100,000 random gene sets outperformed our signature. Since the signature was optimized from this data set, we calculated an additional *p*‐value by determining the fraction of random gene sets that achieved separation within one standard deviation of the signature (Figure 4b). After 1000 repetitions of 1000 permutations, the *p*‐value was 0.005 ± 0.002 (mean ± SD) indicating that not only did the signature isolate the 4T1 samples, but far exceeded random.

Reproducibility and efficacy across disease progression were established by validating the signature by PCR on the scaffolds in a separate cohort of mice at 7, 14, and 21‐days post‐inoculation, which corresponds to the pre‐metastatic niche, early metastatic niche, and metastatic niche, respectively in the 4T1 models.^45^ When considering the individual genes in the signature, each was significantly up‐regulated at day 21 in the 4T1 scaffolds relative to the 67NR and 4T07 scaffolds (Figure 4c). Whereas, at days 7 and 14, almost no individual genes exhibited significance across conditions—the only two exceptions were *Ifitm1* at day 7 and *Slc1a3* at day 14. When the genes were considered as a collective signature, the scaffolds from 4T1 mice were isolated from other tumors at each time point by PCA plots in two dimensions (Figure 4d). Interestingly, on day 7, although the signature isolates 4T1 samples, 4T07 samples are more effectively separated due to the high negative loading of *Ifitm1* in PC1. This trend is consistent with the up‐regulation of interferon‐related signaling in the 4T07 samples (Table S4).

Area under the receiver operating characteristic (ROC) curve (AUC) was calculated for each plot to determine the efficacy of the separation. The distance between each point and the center of the 4T1 centroid on a two‐dimensional PCA plot served as the ranking variable (Figure S8). The AUC was 0.65 (*p* = 0.286), 0.84 (*p* = 0.014), and 0.94 (*p* = 0.001) at days 7, 14, and 21, respectively. Although eigenvalues identified two requisite components for the PCA plots, additional inquiry, using parallel analysis, reported that only one component should be considered for the analysis of day 7 samples (Figure S9). When the AUC was recalculated in one dimension, the signature was able to isolate the 4T1 samples at day 7 with an AUC of 0.94 (*p* = 0.0017). These data validated the signature in a separate cohort with different technology and indicated that the signature successfully isolated metastatic cancer across the spectrum of disease.

### 2.5 Scaffold‐derived signature is specific to the metastatic niche

**FIGURE 5:**
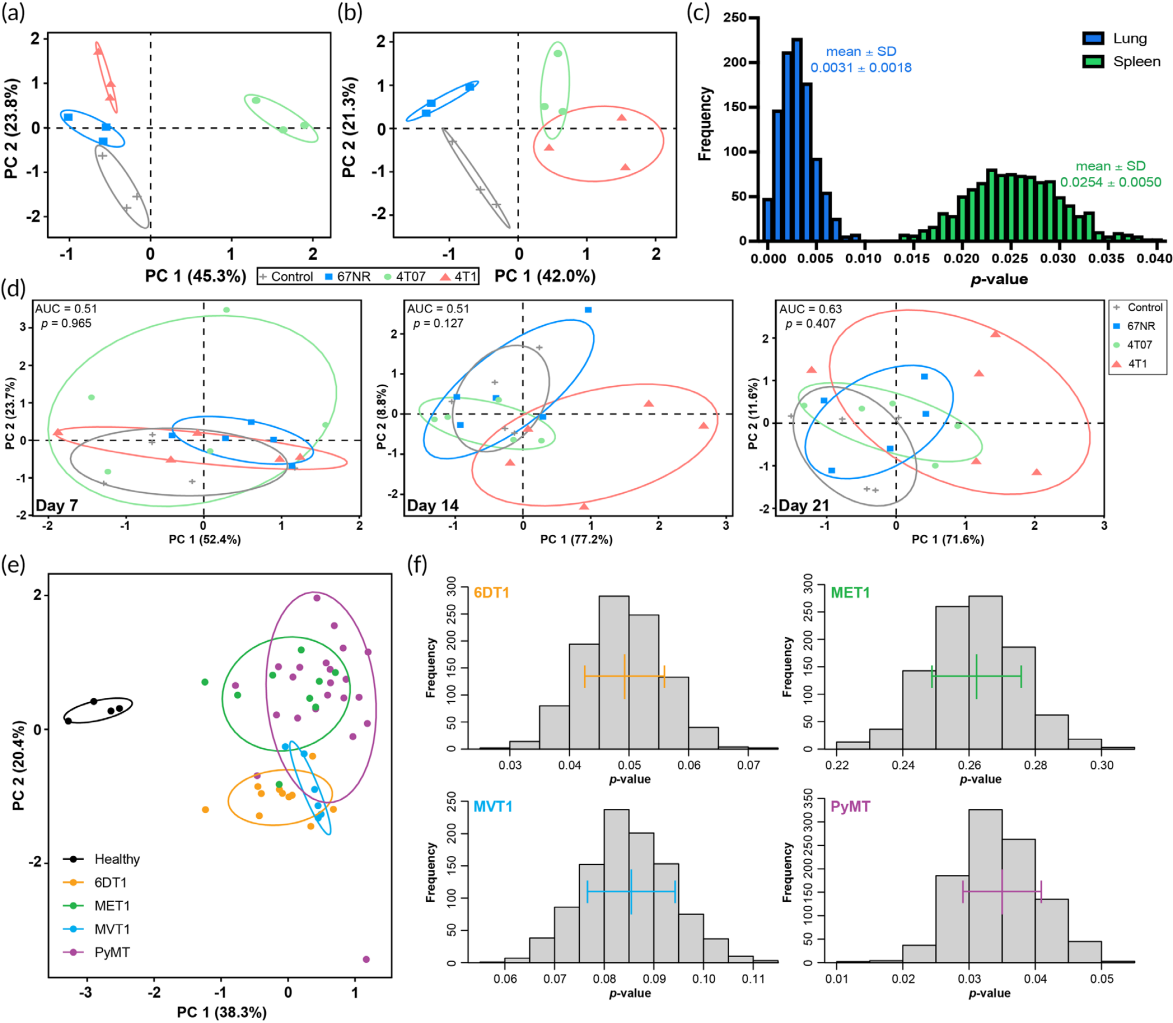
Scaffold‐derived signature was applied to other mouse tissues and models. PCA plots of the signature applied to the (a) lung and (b) spleen RNA‐seq data on day 14, *n* = 3 mice per condition. (c) The *p*‐values were calculated by comparing the separation of the 4T1 cluster against 1000 permutations of 1000 random gene sets. (d) PCA plots of PCR data for the signature on the blood of mice exposed to 67NR, 4T07, and 4T1 cell lines at days 7, 14, and 21, *n* = 5 mice per condition. (e) PCA plot of the signature on RNA‐sequencing data from lungs of FVB/NJ mice found in the GSE150928 data set. Healthy mice (*n* = 4) were compared to transgenic PyMT mice (*n* = 22) and mice inoculated with the 6DT1 (*n* = 11), MVT1 (*n* = 6), and MET1 (*n* = 9) cell lines. (f) The significance of the signature was calculated by comparing the separation of the diseased centroid from the healthy centroid to the separation using 1000 random permutations of genes *(p*‐value) repeated 1000 times (frequency). The *p*‐value indicates the fraction of gene sets that obtained separation greater than the signature. Bars indicate mean ± standard deviation, ellipses correspond to one standard deviation.

The 9‐gene signature was tested on the RNA‐sequencing data from the lung and spleen to investigate its utility in other tissues. The signature was successfully applied to the lung, isolating the 4T1 cluster from the other tissues on a PCA plot with a *p‐*value of 0.0031 (Figure 5a). When the signature was applied to the spleen, the PCA plot could not delineate the 4T07 and 4T1 models (Figure 5b). Even so, the signature outperformed random with a *p*‐value of 0.0254 suggesting the gene expression in the spleen poorly correlates with metastatic potential (Figure 5c). This finding was consistent with the splenic signaling changes (Figure 3b) not reflecting the up‐regulation of key cancer‐related pathways. We also investigated the signature in mouse blood to determine if it could be applied to traditional liquid biopsy (Figure 5d). The signature was unable to fully separate the 4T1 samples from the other conditions at any time point. The most effective separation occurred at day 21 (AUC = 0.63). These data indicate that the signature was not effective on blood samples and emphasize that the information obtained from the scaffold is reflective of the changes occurring at a metastatic site.

### 2.6 The scaffold‐derived signature identifies metastasis in FVB mouse models

We next considered the application of the signature to other mouse models of metastatic breast cancer. The GSE150928 data set in GEO database contained RNA‐sequencing data of lungs from healthy FVB/NJ mice, transgenic PyMT mice, and mice inoculated with the 6DT1, MET1, and MVT1 cell lines. When the signature (*Soga1* was not reported in the source data set) was applied to these models, the diseased lungs clearly grouped together separate from the healthy lungs (Figure 5e, f). The significance was calculated against random, which indicated the signature in the 6DT1 (*p* = 0.049), MVT1 (*p* = 0.085), and PyMT (*p* = 0.035) models was highly effective. Although MET1 lungs successfully separated from healthy lungs, the *p*‐value was 0.262 indicating the signature did not outperform random, possibly due to PI3K‐mediated increased proliferation by MET1 cells.^52^, ^53^

### 2.7 Translational potential of the scaffold‐derived signature

**FIGURE 6:**
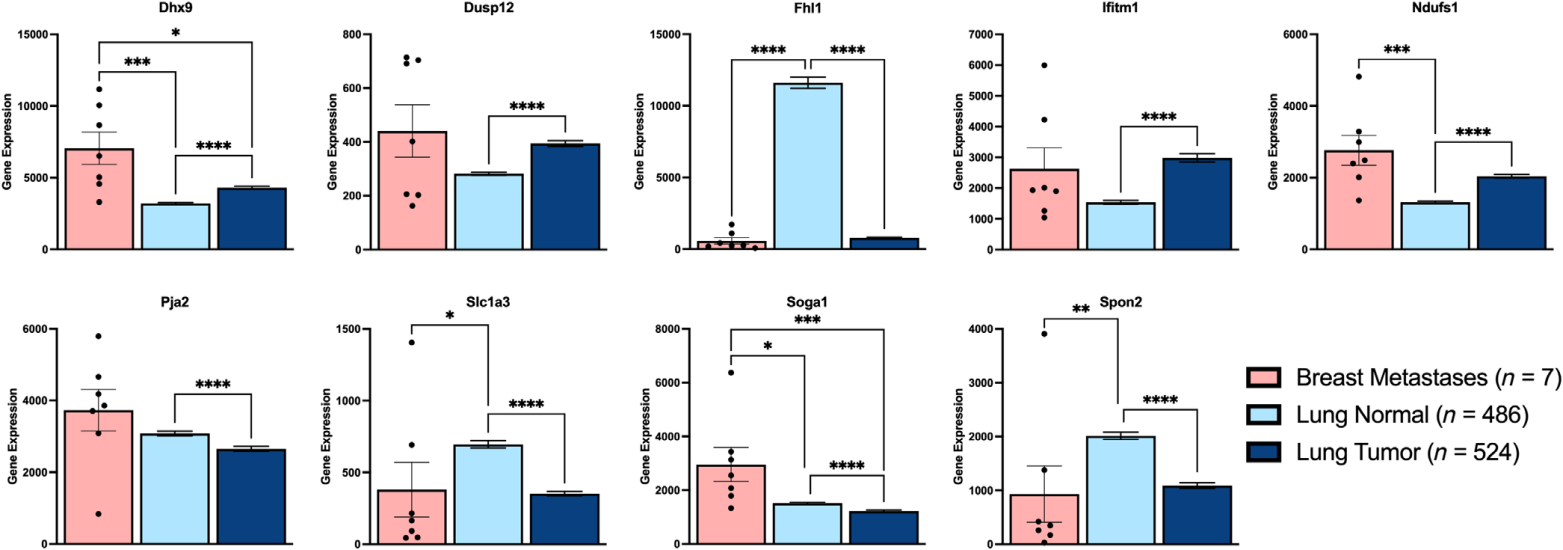
Expression of the genes in the signature in human cancer RNA‐sequencing data available through The Cancer Genome Atlas Research Network. Expression in metastatic tissues in breast cancer (Breast Metastases) was compared to healthy lungs (lung normal) and lungs with a primary tumor (lung tumor), *p < 0.05, **p < 0.01, ***p < 0.001, ****p < 0.0001.

We referenced The Cancer Genome Atlas (TCGA) Research Network (https://www.cancer.gov/tcga) to investigate the application of the scaffold‐derived signature to human metastatic disease.^54^ However, within the database, only seven RNA‐sequencing samples were attributed to metastatic tissues (of unknown source) in breast cancer (Table S5). Since this data was insufficient to draw conclusions in humans, we compared gene expression of the scaffold‐derived signature to that observed within lungs of healthy patients and lung cancer patients. This comparison assumes that the microenvironment of the primary tumor and metastatic lungs may have some similarities. Each gene in the signature was differentially expressed between healthy lungs (lung normal) and lungs with a primary tumor (lung tumor; Figure 6). Furthermore, for 7–9 genes (all but *PJA2* and *SOGA1*), the trends aligned between the diseased lungs and the metastatic breast samples. These data suggest that the signature identified herein may have potential in human cancer diagnostics.

## 3 DISCUSSION

Early detection of metastatic breast cancer has the potential to improve survival outcomes and benefit the millions of women diagnosed with breast cancer each year. In the current clinical paradigm, detection is based on tumor cells, and cancer is only staged as metastatic once a secondary tumor can be macroscopically visualized.^6^ However, by the time tumor cells colonize and proliferate at the distant site, the local microenvironment has experienced myriad changes in cell types, phenotypes, intercellular signaling, and the extracellular matrix that support metastatic outgrowth.^55^ Metastatic formation is dependent on this altered microenvironment, the pre‐metastatic niche and, once this niche is established, metastatic development can continue even after resection of the primary tumor. Diagnostics based on the microenvironment at the pre‐metastatic niche could serve to identify patients at the early stages of metastatic disease.

The 67NR, 4T07, and 4T1 models permit controlled investigations into the microenvironmental changes that correlate with different stages of human disease. Despite the promise such models afford toward understanding the biology of metastatic disease, most efforts comparing these cell lines focus on the analysis of the tumor cells either in cell culture or primary tumors. In vitro, cultures have identified CXCR4 and miR‐200 as potential targets in modulating tumor cell plasticity.^38^, ^39^ Other studies demonstrated that increased metastatic potential correlates with increased adaptability to the microenvironment.^40^, ^41^ However, once these cell lines are inoculated in vivo, analysis of the primary tumor struggles to differentiate metastatic 4T1 cells from the invasive, nonmetastatic 4T07 cells.^35^, ^36^, ^37^ These findings are consistent with our study, wherein the immune cell distribution was highly similar between the 4T07 and 4T1 primary tumors and changes in gene expression correlated with the input cell lines rather than metastatic progression (Figure 1 and S4). The few studies that utilized these cell lines to monitor the biology at metastatic sites did not distinguish 4T07 nonmetastatic and 4T1 metastatic cancer. Walker II et al. reported that cytokine secretion in the spleen, serum, liver, and brain were all comparable between 4T07 and 4T1 mice.^56^ Bosiljcic et al. identified an immunosuppressive role of myeloid‐derived suppressor cells in the lungs of mice treated with both cell lines.^57^ We were able to advance this research and successfully delineate the 4T07 and 4T1 cell lines using the host biology through the combination of scaffolds and gene expression.

The nine genes identified in our signature (*Dhx9*, *Dusp12*, *Fhl1*, *Ifitm1*, *Ndufs1*, *Pja2*, *Slc1a3*, *Soga1*, and *Spon2*) have each been investigated for their role in cancer. Seven of the genes are reported to have primarily pro‐cancer functions. *Dhx9* (DExH‐Box Helicase 9; UniProt Q08211), *Dusp12* (Dual Specificity Phosphatase 12; UniProt Q9UNI6), and *Spon2* (Spondin 2; UniProt Q9BUD6) act as oncogenes and are strongly associated with tumorigenesis.^58^, ^59^, ^60^ *Ifitm1* (Interferon Induced Transmembrane Protein 1; UniProt P13164) has been directly tied to aggressiveness of breast cancer cells.^61^ *Slc1a3* (Solute Carrier Family 1 Member 3; UniProt P43003) and *Soga1* (Suppressor of Glucose, Autophagy Associated 1; UniProt O94964) enhance tumor metabolism and are associated with poor prognosis.^62^, ^63^ *Ndufs1* (NADH:Ubiquinone Oxidoreductase Core Subunit S1; UniProt P28331) is involved in the adaptability of tumor cells to their environment, particularly at low pH.^64^ Furthermore, *Dhx9*, *Dusp12*, *Fhl1*, and *Ifitm1* are each correlated with the invasion of tumor‐infiltrating immune cells into the microenvironment.^59^, ^65^, ^66^, ^67^ In contrast, *Pja2* (Praja Ring Finger Ubiquitin Ligase 2; UniProt O43164) promotes anti‐cancer M1 macrophage polarization.^68^ Interestingly, *Fhl1* (Four and a Half LIM Domains 1; UniProt Q13642) suppresses tumor growth, yet the phosphorylated protein acts as a tumor promoter.^69^ Since the data collected herein is at the genomic level, we cannot discern the extent of phosphorylation in our system.

Gene signatures derived from the primary tumor or blood have quickly become a popular approach to cancer research.^6^ However, these human‐derived signatures are limited by clinically available data. Diagnostic signatures focus on the extremes (healthy vs. cancer, stage I vs. stage IV) to increase efficacy.^12^, ^13^, ^14^, ^15^ Prognostic signatures are developed across the disease spectrum and are often as correlated with the stage of disease as they are with gene expression.^70^, ^71^, ^72^, ^73^, ^74^, ^75^ In fact, most breast cancer signatures that claim to have clinical value do not outperform random gene sets and are rather artifacts of increased proliferation in cancer.^51^, ^52^ When a selection of human gene signatures was applied to lungs in our system, each failed to match the ability of our signature to delineate metastatic potential against random gene sets (Figure S10).^70^, ^71^, ^73^, ^74^, ^75^, ^76^ We generated a 9‐gene signature that separates metastatic breast cancer from healthy, noninvasive, and invasive nonmetastatic counterparts (Figure 4). Our signature outperforms random gene sets, applies across the entire disease spectrum, is effective in independent mouse studies (Figure 5), and each gene is differentially expressed in human lung cancer (Figure 6).

In conclusion, this unique signature effectively identifies metastatic disease from invasive, nonmetastatic disease prior to the formation of a secondary tumor. Currently, at early time points (days 7 and 14) both the 4T07 and 4T1 models would be diagnosed as regional breast cancer (Stage II/III) since tumor cells are found in the lymph nodes, but there are no detectable secondary tumors.^77^ The engineered metastatic niche provides a platform for diagnosing metastatic breast cancer without secondary tumor formation. The use of the signature with the engineered niche has utility in initial staging of disease and could further be applied after the resection of the primary tumor to analyze for disease recurrence. The reported methodology and signature could be highly effective in a translational setting and would be important to evaluate in a clinical study that would incorporate greater genetic variation and patient factors. Furthermore, the methods outlined herein are not limited to breast cancer and could be applied to identify similar signatures for other cancer types. Given the toxic nature of cancer therapies, it is important to administer these drugs to patients who truly need them, avoiding devastating side effects in the nearly 30% of all women who will be diagnosed with nonmetastatic breast cancer in their lifetime.

## 4 MATERIALS AND METHODS

All materials were obtained from Thermo Fisher Scientific (Waltham, MA) unless otherwise stated.

### 4.1 Scaffold fabrication

Porous, polycaprolactone scaffolds were constructed as previously described^25^, ^26^, ^27^, ^28^ (Figure S11). Briefly, ester‐terminated polycaprolactone (Evonik; Essen, Germany) was mixed with sodium chloride (250–425 μm) at a ratio of 1:30 at 85°C for 30 min. Once the mixture cooled to room temperature, 77.5–80.0 mg of the solution was pressed into 5 mm × 2 mm (*d* × *h*) disks using a hydraulic press at 1000 psi for 45 s. Scaffolds were heated at 60°C for 5 min per side to form a continuous polymer structure. Subsequently, scaffolds were incubated in deionized water for 1.5 h at room temperature to leach out the salt, sterilized in 70% ethanol, and stored at −80°C until use.

### 4.2 Cell lines and culture

The 67NR (nonmetastatic), 4T07 (micro‐metastatic), and 4T1 (metastatic) murine triple‐negative breast cancer cell lines were derived from a single spontaneous tumor in a female BALB/cfC3H mouse,^32^, ^33^, ^34^ with female mice used due to breast cancer having a greater incidence in women. The 67NR and 4T07 cell lines were purchased from the Karmanos Institute at Wayne State University. The 4T1 cell line was obtained from Perkin Elmer (Waltham, MA). Cells were maintained in high glucose DMEM supplemented with 10% (v/v) fetal bovine serum (VWR; Radnor, PA), 2 mM L‐glutamine, and 1 mM mixed nonessential amino acids.

### 4.3 Animal studies

All animal studies were conducted with approval from and in accordance with the University of Michigan Institutional Animal Care and Use Committee guidelines and protocols (PRO00009715). Scaffolds were implanted subcutaneously into female BALB/c mice (Jackson Laboratory; Bar Harbor, ME) at 7–8 weeks‐old as previously described^25^, ^26^, ^27^, ^28^, ^31^ (Figure S11). Mice were anesthetized via 2% isoflurane and provided carprofen analgesia (5 mg/kg, subcutaneous injection). Scaffolds were implanted through a small incision made on the upper back of the mice, which was sealed with sterile wound clips (Reflex 7 mm). Scaffolds were allowed to equilibrate in the subcutaneous space for 2 weeks before the mice received 50 μL orthotopic inoculations of 67NR, 4T07, or 4T1 cells at a concentration of 40 million cells/mL phosphate buffered saline (PBS) in the fourth, right mammary fat pad (2 million cells/mouse).^25^, ^26^, ^27^, ^31^, ^45^ Tumors were allowed to progress for 7‐, 14‐ or 21‐days post‐inoculation. Healthy mice without tumors acted as controls. Animal cages were randomly assigned to cell lines and time points prior to tumor inoculation.

### 4.4 Flow cytometry

Lungs, scaffolds, primary tumors, and spleens were explanted from mice either 7‐ or 14‐days after tumor inoculation for flow cytometry.^25^, ^28^, ^31^ Tissues were extracted from healthy mice at each time point to as controls. Lungs, scaffolds, and primary tumors were minced and digested with Liberase (Roche; Basel, Switzerland). All tissues were processed through a 70 μm filter to a single cell suspension, washed with MACS buffer (PBS containing 0.5% (v/v) bovine serum albumin and 2 mM EDTA), blocked with an anti‐mouse CD16/CD21 antibody, and stained with conjugated primary antibodies to identify immune cell populations. Samples were processed on a ZE5 Cell Analyzer (Bio‐Rad; Hercules, CA) and analyzed with FlowJo (BD; Franklin Lakes, NJ).

Immune cells were defined by expression of anti‐mouse CD45 (AF700; Biolegend; San Diego, CA). Immune cell populations were identified using anti‐mouse antibodies (Biolegend) in two panels. Panel 1 (innate immunity) included CD11b (BV510), F4/80 (PECy7), Gr1 (PacBlue), Ly6C (PE), and CD11c (APC). Panel 2 (adaptive immunity) included CD4 (BV510), CD8 (FITC), CD19 (PacBlue), and CD49b (PECy7). Cell types were classified as follows: macrophages (CD11b + F4/80+), neutrophils (CD11b + Gr1+), monocytes (CD11b + Ly6C+), dendritic cells (CD11c + F4/80‐), CD4+ T Cells (CD4+), CD8+ T Cells (CD8+), B Cells (CD19+), and NK Cells (CD49b+).

### 4.5 Library preparation and RNA‐sequencing

Lungs, scaffolds, primary tumors, and spleens were explanted from mice 14‐days after tumor inoculation for RNA‐sequencing.^26^, ^27^ Lungs, scaffolds, and spleens were simultaneously extracted from healthy mice. Tissues were immediately flash‐frozen in isopentane. Frozen tissues were transferred to new tubes containing TRIzol and immediately homogenized. Samples were centrifuged at 10,000 × *g* for 10 min and the supernatant was collected for RNA extraction via the Rneasy® Mini kit (Qiagen; Hilden, Germany). RNA was eluted into nuclease‐free deionized water and submitted to the University of Michigan Advanced Genomics Core for sequencing. Quality control was completed with an Agilent TapeStation (Santa Clara, CA). Library preparation was conducted using QuantSeq (Lexogen; Vienna, Austria) and the samples were sequenced on a NovaSeq (SP) 100 cycle (Illumina; San Diego, CA) with an estimated 10 million reads/sample.

The raw gene count matrices for each tissue acted as inputs for DESeq2 in R.^78^ Genes with a count of 10 or under across all samples were filtered out. DESeq2 was used to normalize gene counts and identify differentially expressed genes between each condition (control, 67NR, 4T07, 4T1) within a given tissue. The mixOmics package in R^50^ was used to identify an initial gene set for the determination of tissue‐specific signatures. The members of the signatures were further narrowed down through polymerase chain reaction (PCR) validation.

### 4.6 Gene set enrichment analysis (GSEA)

All mouse genes were converted to their human orthologs using biomaRt.^79^, ^80^ The normalized tables outputted from DESeq2 (with human genes) were used as the expression datasets for GSEA. Gene sets were selected from the hallmark, curated, and gene ontology databases. Other GSEA parameters were set to 1000 permutations, with no collapse, with a permutation type of “gene_set.” Up‐ and down‐regulated pathways were identified between the conditions within each tissue independently.

### 4.7 PCR testing

Lungs, scaffolds, and blood were extracted from mice at 0‐ (healthy), 7‐, 14‐, and 21‐days after tumor inoculation. Lungs and scaffolds were immediately flash‐frozen with isopentane and RNA was collected as described for RNA sequencing. Blood was collected by cardiac puncture into tubes containing 25 mM ETDA. Blood RNA was immediately isolated using a Mouse RiboPure™‐Blood RNA Isolation Kit. The concentration and quality of RNA were measured by a Nanodrop 2000. RNA was converted to cDNA with the iScript™ cDNA Synthesis Kit (Bio‐Rad). PCR was conducted using the QuantiTect SYBR Green master mix (Qiagen).^26^, ^81^ Briefly, 0.5 μg cDNA in SYBR Green was added to the bottom of a 384‐well plate. Forward and reverse primers, diluted to 1 μM, were layered on top of the cDNA (Table S6). Plates were sealed and read on a QuantStudio 5 using the program for ∆∆*C*_*T*_. The outputs were analyzed with the Design & Analysis Software (v2.6.0) to obtain *C*_*q*_ values. Fold changes of tumor‐bearing animals relative to the healthy controls were obtained using *Polr2a*, *Ubc*, and *Ywhaz* as endogenous controls.

### 4.8 External data sets

The translatability of the scaffold‐derived signature to other mouse and human models was conducted using publicly available datasets. Normalized RNA‐sequencing data of cultured 67NR, 4T07, and 4T1 cell lines and lungs in FVB/NJ mouse models of metastatic breast cancer were obtained from the Gene Expression Omnibus (GEO) series GSE150928 with alignment to the mm9 mouse genome.^82^ Healthy lungs from mice were compared to lungs from transgenic MMTV‐PyMT mice and from mice that received orthotopic inoculations of 6DT1, MET1, or MVT1 metastatic breast cancer cell lines. Human RNA‐sequencing data from normal lung, lung tumors, and metastatic breast cancer tissues was obtained using the TMNplot tool, where the developers pre‐analyzed data from over 11,000 TCGA files.^54^

### 4.9 Statistical analysis

Statistical significance between the immune cell populations and PCR gene expression was determined using two‐way ANOVA with Tukey's correction for multiple hypothesis testing using GraphPad Prism 9. Significance of the TCGA data was determined with a Kruskal–Wallis test assuming non‐Gaussian distributions using GraphPad Prism 9. Hierarchical clustering of heatmaps was completed using the “complete” agglomeration method in R. Adjusted *p*‐values of differential gene expression were calculated with DESeq2 using the Wald test with the Benjamini and Hochberg correction for multiple hypothesis testing. Significance of signaling pathways were determined using GSEA. ROC curves were generated by calculating the distance from each point on the PCA plot to the center of the 4T1 centroid, where 4T1 samples were positive cases and control, 67NR, and 4T07 samples were negative cases. The number of principal components (PCs) used for this calculation was determined with eigenvalues (using the Kaiser rule in Prism), which selected two components for each plot.

To calculate the significance of the signatures, PCA was run on 1000 random permutations of genes (of the same size as the signature). For each permutation, we calculated the average Euclidean distance between the 4T1 samples and the other conditions using PC1 and PC2 as the axes. The *p*‐value was calculated as the fraction of permutations where the 4T1 samples had no overlap and had an average distance within one standard deviation of the signature (see Supplementary Material for further details). This process was repeated 1000 times. Significance of the scaffold signature on the lungs was calculated through the same methods, with the *p*‐value defined as the fraction of random permutations with an average separation distance greater than the signature. Figures with error bars depict mean ± SEM with α = 0.05 unless otherwise stated.

## 5 CONCLUSIONS

Using an engineered metastatic niche, we identified a 9‐gene signature (*Dhx9*, *Dusp12*, *Fhl1*, *Ifitm1*, *Ndufs1*, *Pja2*, *Slc1a3*, *Soga1*, *Spon2*) that successfully delineated metastatic breast cancer from nonmetastatic breast cancers including an invasive cell line without metastatic potential. This signature was not successful in traditional liquid biopsy but was highly efficient in the native metastatic site (lung), emphasizing the importance of tissue analysis in cancer diagnostics. Each gene was identified in the human transcriptome and differentially expressed in lung cancer. The reported signature combined with engineered niche could be highly effective in a clinical setting, where preventing over‐ and under‐treatment remains a key challenge.

## AUTHOR CONTRIBUTIONS

**Sophia Orbach:** Conceptualization (lead); data curation (lead); formal analysis (lead); funding acquisition (supporting); investigation (lead); methodology (equal); visualization (lead); writing – original draft (lead); writing – review and editing (equal). **Christian DeVaull:** Formal analysis (supporting); investigation (supporting). **Elizabeth Bealer:** Investigation (supporting); writing – review and editing (supporting). **Brian Ross:** Investigation (supporting); writing – review and editing (supporting). **Jacqueline Jeruss:** Funding acquisition (supporting); supervision (supporting). **Lonnie Shea:** Conceptualization (supporting); funding acquisition (lead); methodology (equal); supervision (lead); writing – review and editing (equal).

## CONFLICT OF INTEREST STATEMENT

Lonnie D. Shea and Jacqueline S. Jeruss have pending patent applications US20170281798A1 (Northwestern University) and US2017012556 (University of Michigan) with the scaffold technology. The remaining authors have no conflicts of interest to declare.

## Supporting information


**Data S1:** Supporting information

**Data S2:** Supporting information

## Data Availability

The RNA‐sequencing data that support these findings are openly available in the NCBI GEO repository, accession GSE233001. Data not included in the manuscript or supplementary material are available from the authors upon request.
