# Supplementary material for: An engineered niche delineates metastatic potential of breast cancer
Source: Bioeng Transl Med. 2023 Sep 29;9(1):e10606. doi: 10.1002/btm2.10606 (PMC10771563; doi:10.1002/btm2.10606)
Supplement: Supplementary file 1 — Data S1: Supporting information [file BTM2-9-e10606-s002.docx]

**Supplementary Material**

Code used to calculate p-value of signatures using comparisons to random gene sets

**File 1: wrapper.R**

*#Load packages*

library(dplyr)

library(sf)

*#Call functions*

source("~/p.function.R")

*#Read in normalized RNA data, identify sample names, and input signature genes*

data <- read.csv('normalized.scaf.csv', header = T, row.names = 1)

sample <- rep(c('Control', '67NR', '4T07', '4T1'), each = 3)

sig_genes <- c('Dhx9', 'Dusp12', 'Fhl1', 'Ifitm1', 'Ndufs1', 'Pja2', 'Slc1a3', 'Soga1', 'Spon2')

*#Calculate the distance between the 4T1 data points and the control, 67NR, and 4T07data points on a PCA #plot using PC1 and PC2*

*#Store the average distance between clusters (sig_dist) and distance minus one standard deviation (one_sd)*

dist <- sig.p(data, sample)

sig_dist <- dist[[1]]

one_sd <- dist[[2]]

*#Calculate the fraction of random gene sets with distance greater than the signature (third term = sig_dist) or*

*#within one standard deviation of the signature (thid term = one_sd)*

results <- p.value(data, sample, sig_dist)

*#Save and visualize p-values*

write.csv(results, 'p.values.csv')

hist(results)

**File 2: p.function.R**

*#Calculate the distance between the 4T1 data points and the control, 67NR, and 4T07 data points on a PCA #plot using PC1 and PC2*

*#To calculate the standard deviation, the distance is calculated between each pair of 4T1 points with the #control, 67NR, and 4T07 points*

sig.p <- function(tissue, sample) {

*#Subset RNA-seq data to isolate signature*

data <- t(tissue[sig_genes,])

*#Run PCA*

pca1 <- prcomp(data, scale. = T)

*#Identify the (PC1, PC2) coordinates for each sample*

control <- pca1$x[sample == 'Control', 1:2]

nr <- pca1$x[sample == '67NR', 1:2]

t07 <- pca1$x[sample == '4T07', 1:2]

t1 <- pca1$x[sample == '4T1', 1:2]

*#Calculate the distance between each 4T1-control pair*

control_dist <- matrix(, nrow = 3, ncol = 3)

for (i in 1:3) {

for (j in 1:3) {

control_dist[i,j] <- sqrt(sum((matrix(control[i,]) - matrix(t1[j,]))^2))

}

}

*#Calculate the distance between each 4T1-67NR pair*

nr_dist <- matrix(, nrow = 3, ncol = 3)

for (i in 1:3) {

for (j in 1:3) {

nr_dist[i,j] <- sqrt(sum((matrix(nr[i,]) - matrix(t1[j,]))^2))

}

}

*#Calculate the distance between each 4T1-4T07 pair*

t07_dist <- matrix(, nrow = 3, ncol = 3)

for (i in 1:3) {

for (j in 1:3) {

t07_dist[i,j] <- sqrt(sum((matrix(t07[i,]) - matrix(t1[j,]))^2))

}

}

*#Calculate the average distance between the 4T1 clusters*

sig_dist <- mean(c(control_dist, nr_dist, t07_dist))

*#Calculate the standard deviation of all distances*

sd_dist <- sd(c(control_dist, nr_dist, t07_dist))

one_sd <- sig_dist - sd_dist

*#Return average distance (sig_dist) and average distance minus one standard deviation (one_sd)*

out <- list(sig_dist, one_sd)

return(out)

}

*#Calculate the fraction of random gene sets with distance greater than the signature*

*#To expedite run, distances were calculated between the centroids of each cluster*

p.value <- function(tissue, sample, dist) {

p <- matrix(, nrow = 1000, ncol = 1)

for (j in 1:1000) {

results <- matrix(, nrow = 1000, ncol = 1)

for (i in 1:1000) {

*#Subset RNA-seq data with random sampling of 9 genes (# genes in signature)*

data <- t(sample_n(tissue, 9))

*#Run PCA*

pca1 <- prcomp(data, scale. = T)

*#Calculate the centroid (PC1, PC2) of each group of cells on the PCA plot*

control <- pca1$x[sample == 'Control', 1:2]

control <- rbind(control, control[1,])

control = st_polygon(list(control))

control_center <- st_centroid(control)

nr <- pca1$x[sample == '67NR', 1:2]

nr <- rbind(nr, nr[1,])

nr = st_polygon(list(nr))

nr_center <- st_centroid(nr)

t07 <- pca1$x[sample == '4T07', 1:2]

t07 <- rbind(t07, t07[1,])

t07 = st_polygon(list(t07))

t07_center <- st_centroid(t07)

t1 <- pca1$x[sample == '4T1', 1:2]

t1 <- rbind(t1, t1[1,])

t1 = st_polygon(list(t1))

t1_center <- st_centroid(t1)

*#Calculate the average distance between the 4T1 samples and the control, 67NR, and 4T07 samples*

control_dist <- sqrt(sum((matrix(t1_center) - matrix(control_center))^2))

nr_dist <- sqrt(sum((matrix(t1_center) - matrix(nr_center))^2))

t07_dist <- sqrt(sum((matrix(t1_center) - matrix(t07_center))^2))

distance <- mean(c(control_dist, nr_dist, t07_dist))

*#Return TRUE if the calculated distance is greater than the signature (better separation)*

*#Return FALSE if the calculated distance is smaller than the signature (worse separation)*

results[i] <- all(distance >= dist)

}

print(j)

*#Calculate the fraction of random gene sets with better separation than the signature*

p[j] <- length(results[results == TRUE])/1000

}

return(p)

}


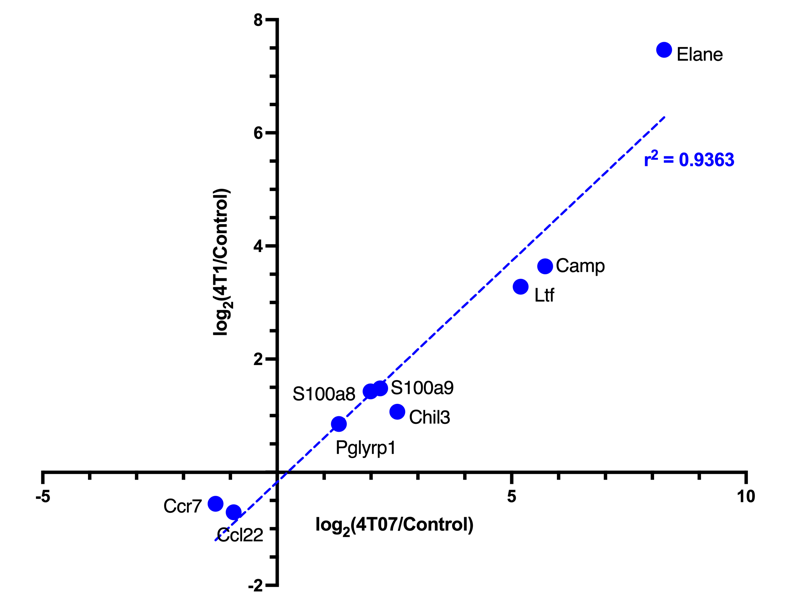


**Figure S1:** Correlation of 4T07 and 4T1 gene expression in the scaffolds for the signature identified by Oakes, R.S. *et al*. (doi: 10.1158/0008-5472.CAN-19-1932). Nine out of ten genes were found in our data set, with *Bmp15* not present. These genes, enriched in neutrophils and MDSCs, were highly correlated between 4T07 and 4T1 scaffolds relative to healthy controls.


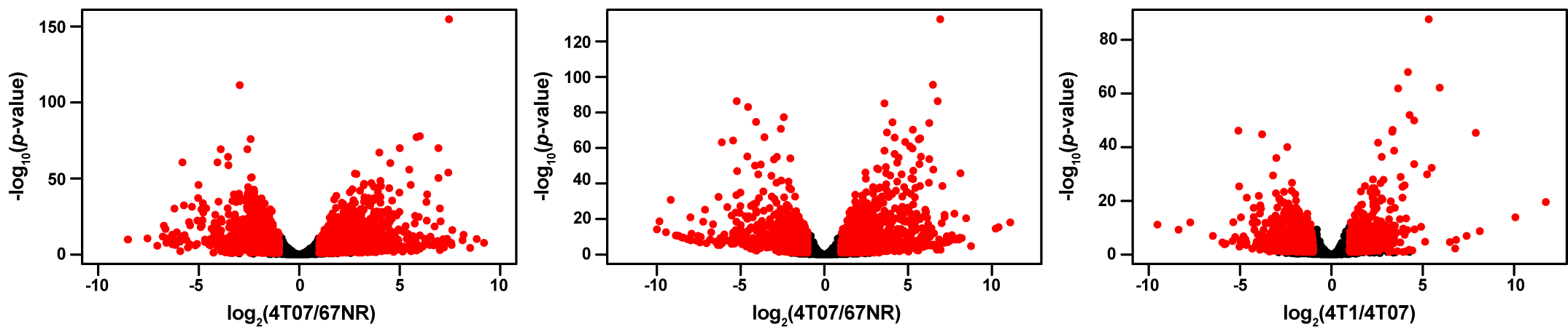


**Figure S2:** Volcano plots of the differentially expressed genes between each primary tumor pair. Red dots correlate to genes with adjusted *p*-values < 0.05 and fold change > 2.0.


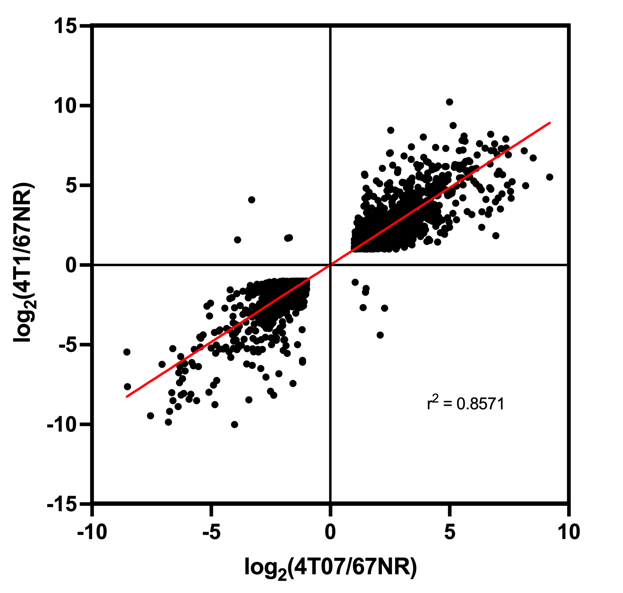


**Figure S3:** Correlation of differentially expressed genes in 4T07 and 4T1 primary tumors relative to 67NR.


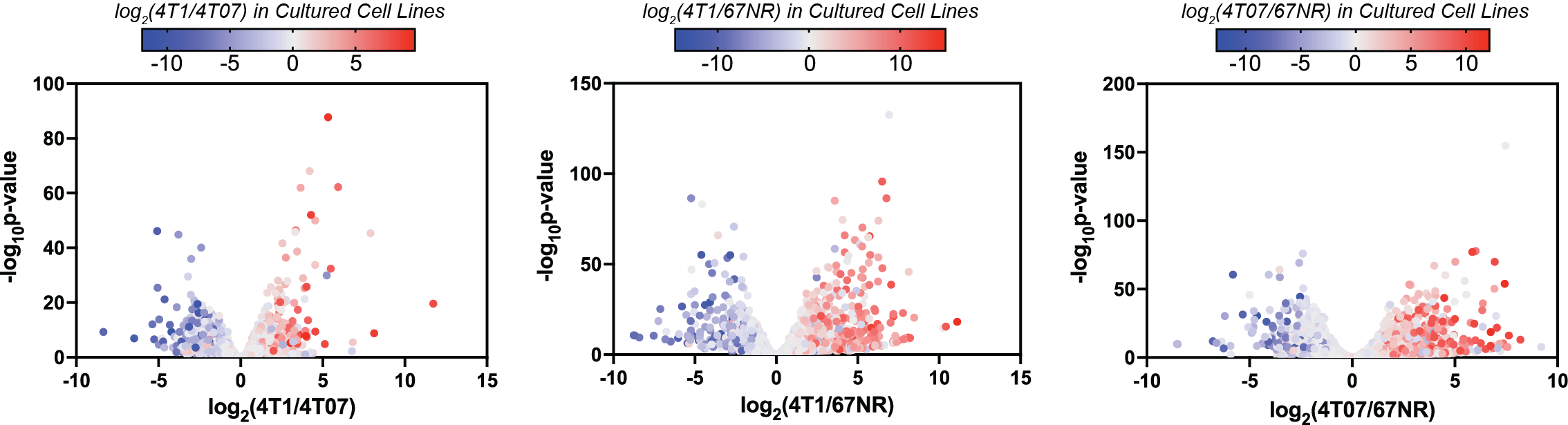


**Figure S4:** Volcano plots comparing each pair of primary tumors. Color scheme correlates with the fold change of gene expression in the cultured cell lines as reported in the GSE150928 data set.


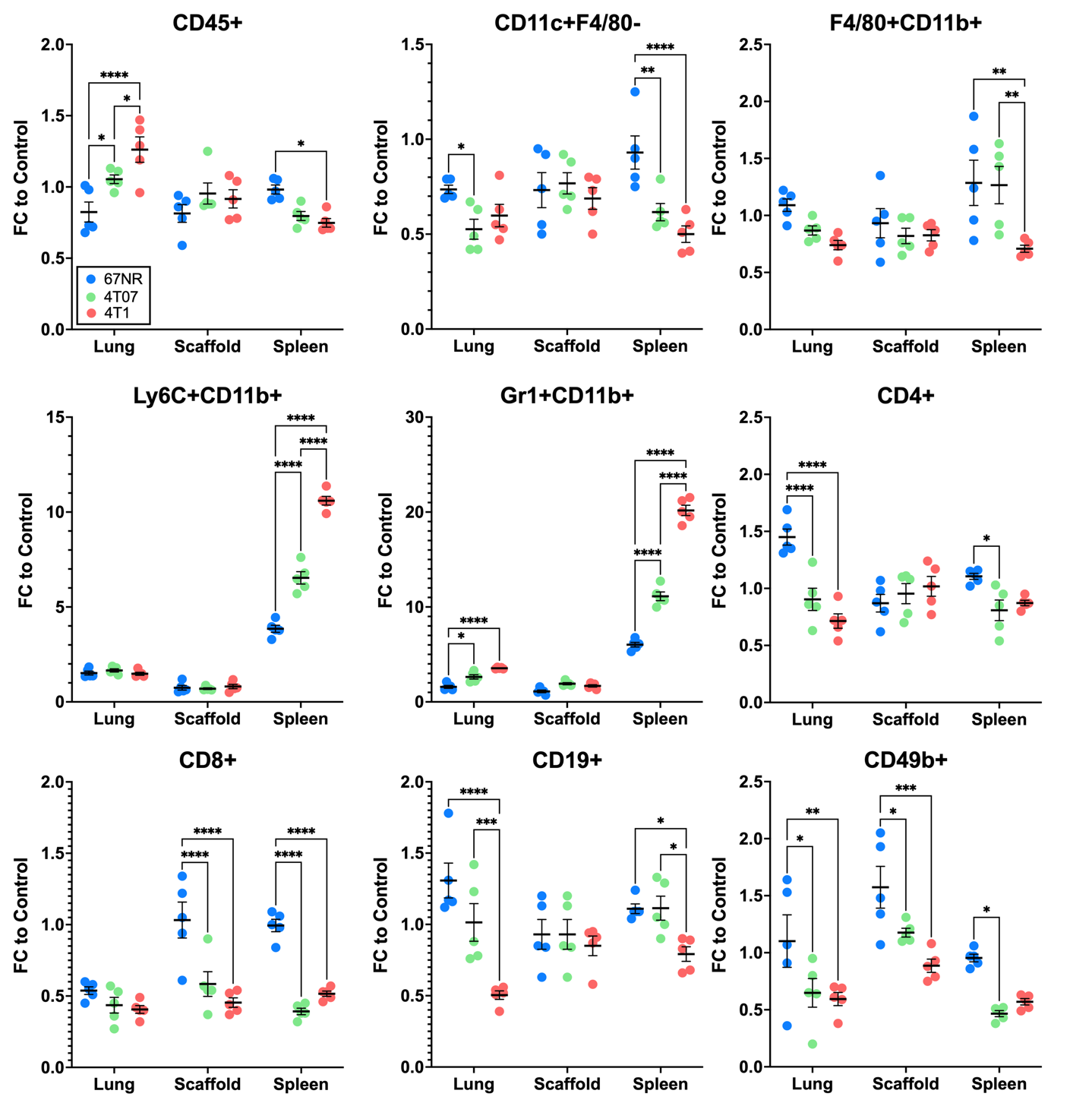


**Figure S5:** Diseases-induced changes in immune cell populations in the lung, scaffold, and spleen on day 14. Populations included all immune cells (CD45+), dendritic cells (CD11c+F4/80-), macrophages (F4/80+CD11b+), monocytes (Ly6C+CD11b+), neutrophils (Gr1+Cd11b+), CD4+ T cells (CD4+), CD8+ T cells (CD8+), B cells (CD19+), and NK cells (CD49b+), *n* = 5 mice per condition, **p* < 0.05, ***p* < 0.01, ****p* < 0.001, *****p* < 0.0001.


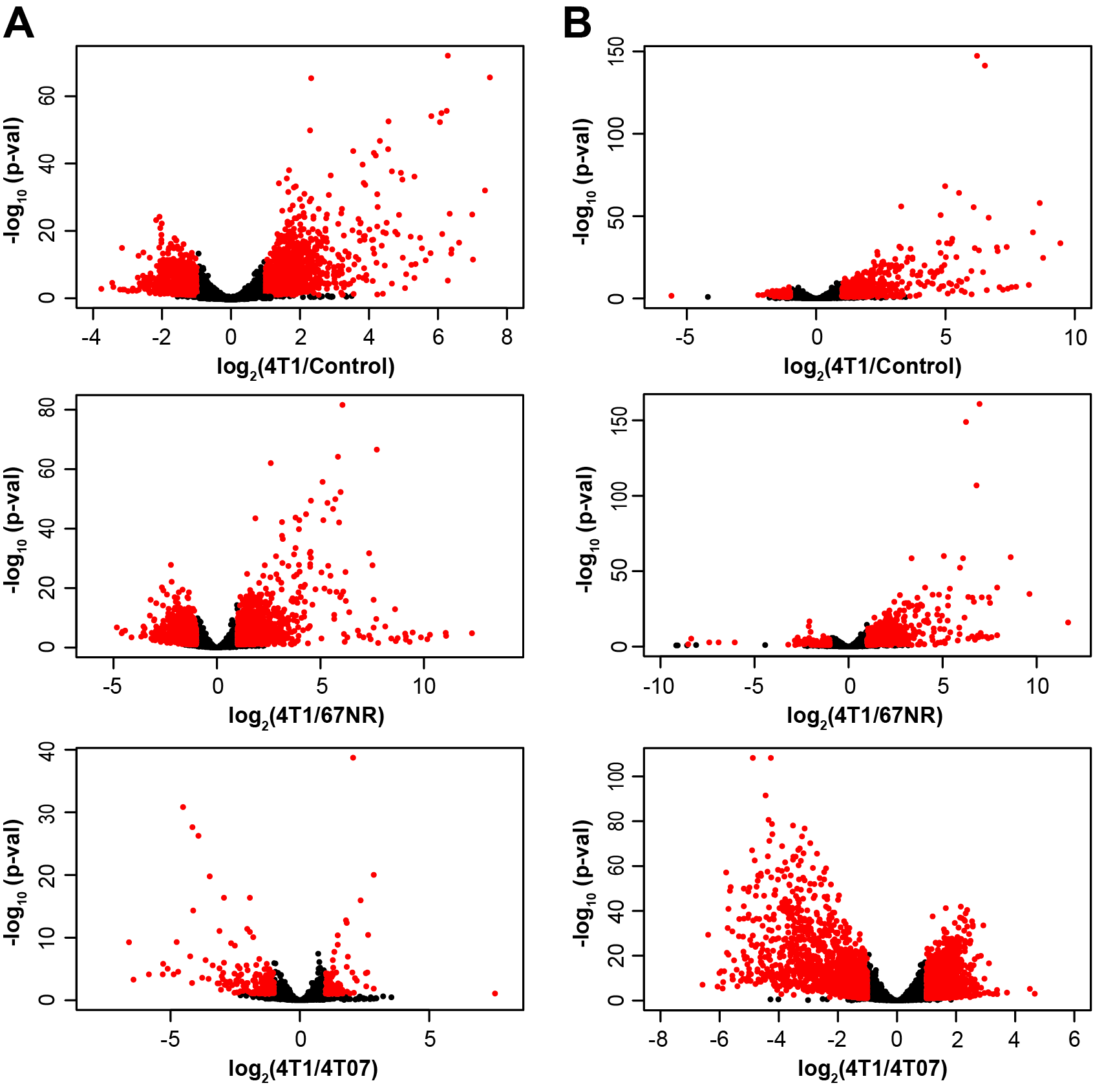


**Figure S6:** Differentially expressed genes between 4T1 samples and the other conditions in the (**A**) spleen and (**B**) lung. Red denotes genes with fold change > 2.0 and *p*-value < 0.10.


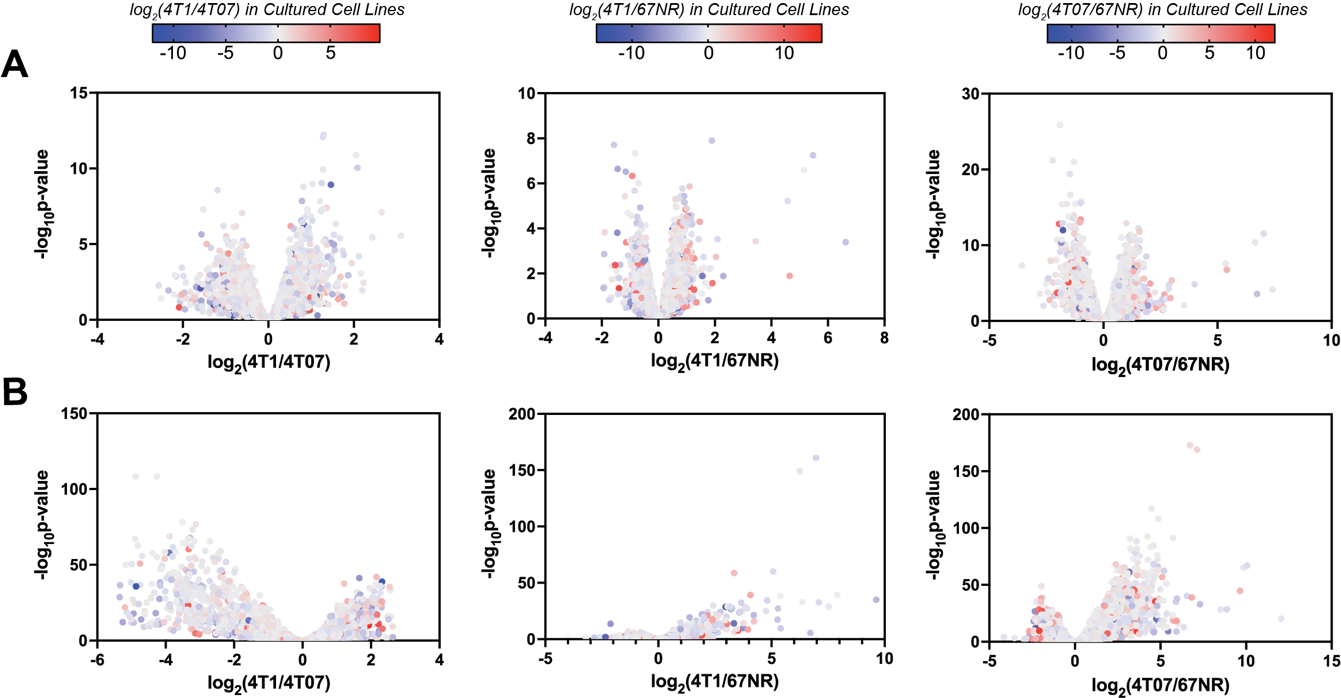


**Figure S7:** Volcano plots comparing each diseased pair in the (**A**) scaffold and (**B**) lung. Color scheme correlates with the fold change of gene expression in the cultured cell lines as reported in the GSE150928 data set.


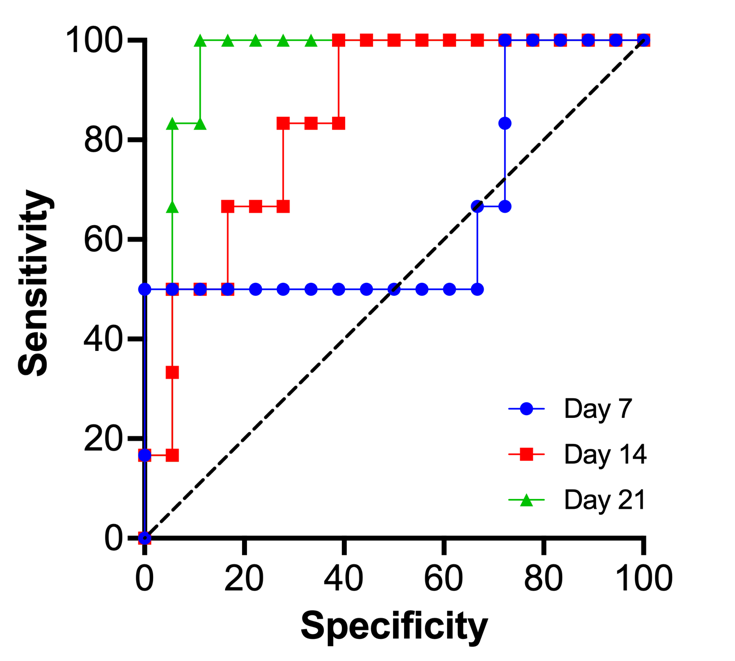


**Figure S8:** Sensitivity-specificity plots were used as receiver operating characteristic curves (ROC) using PC1 and PC2 as coordinates. Points were ranked by the distance from the centroid of the 4T1 cluster, where 4T1 samples were considered positives and all other samples (control, 67NR, 4T07) were considered negatives.


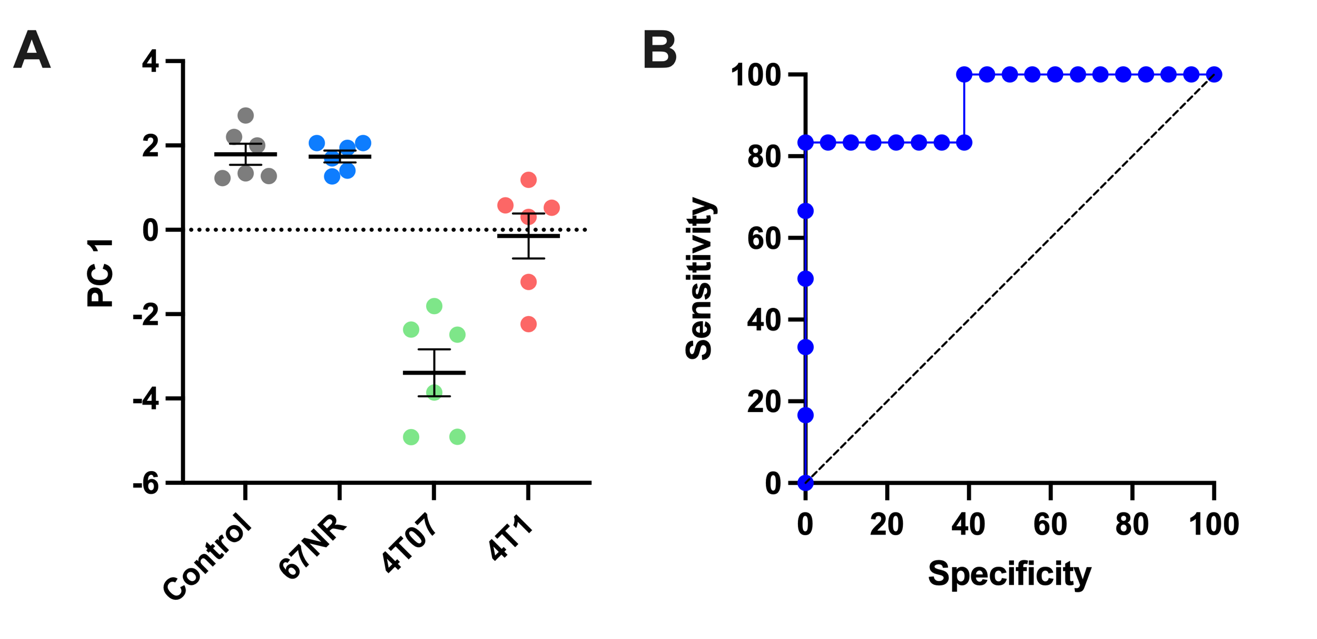


**Figure S9:** Signature validation at day 7 using one principal component. (**A**) Plot of PC1 coordinates. (**B**) Sensitivity-specificity plot where points were ranked by the distance from the average of the 4T1 cluster, where 4T1 samples were considered positives and all other samples (control, 67NR, 4T07) were considered negatives.

**Supplementary Figure S10:** The reported signature outperforms published gene signatures on clinical breast cancers at differentiating metastatic potential in the mouse lungs^1-6^. The AUC identifies the separation of 4T1 lungs from control, 67NR, and 4T07 lungs. The p-value detects how well the signatures outperform random gene sets of the same size.


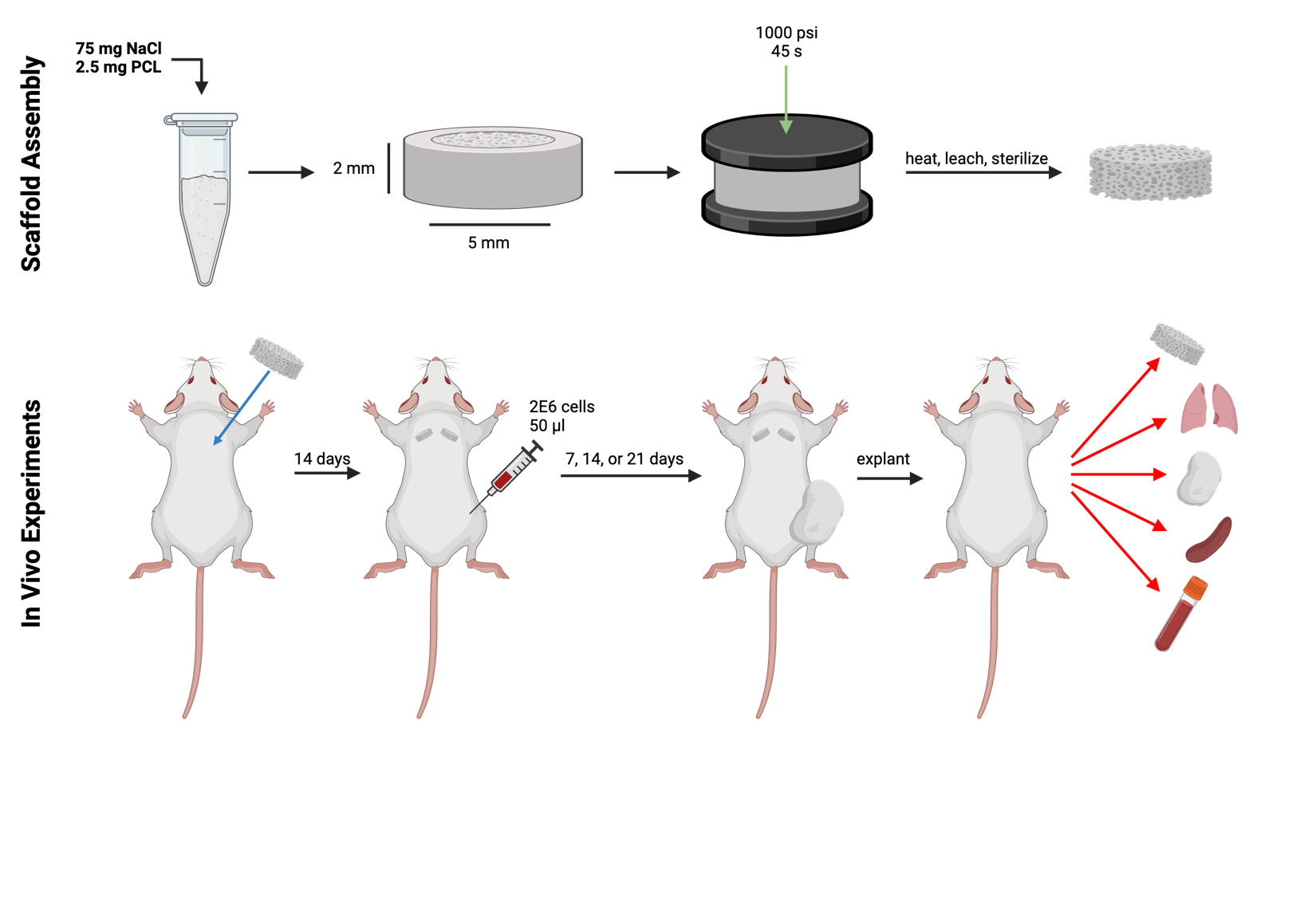


**Figure S11:** Experimental schematic.

**References**

**1.** Li Y, Feng J, Wang T, et al. Construction of an immunogenic cell death-based risk score prognosis model in breast cancer. *Front Genet.* 2022;13:1069921.

**2.** Ma J, Chen C, Liu S, et al. Identification of a five genes prognosis signature for triple-negative breast cancer using multi-omics methods and bioinformatics analysis. *Cancer Gene Ther.* 2022;29(11):1578-1589.

**3.** Shimizu H, Nakayama KI. A 23 gene-based molecular prognostic score precisely predicts overall survival of breast cancer patients. *EBioMedicine.* 2019;46:150-159.

**4.** Yang YS, Ren YX, Liu CL, et al. The early-stage triple-negative breast cancer landscape derives a novel prognostic signature and therapeutic target. *Breast Cancer Res Treat.* 2022;193(2):319-330.

**5.** Zhang J, Pan S, Han C, et al. Combination of Immune-Related Network and Molecular Typing Analysis Defines a Three-Gene Signature for Predicting Prognosis of Triple-Negative Breast Cancer. *Biomolecules.* 2022;12(11).

**6.** Zou Y, Xie J, Zheng S, et al. Leveraging diverse cell-death patterns to predict the prognosis and drug sensitivity of triple-negative breast cancer patients after surgery. *Int J Surg.* 2022;107:106936.
